# Visual attention span as a predictor of reading fluency and reading comprehension in Arabic

**DOI:** 10.3389/fpsyg.2022.868530

**Published:** 2022-11-22

**Authors:** Faris H. R. Awadh, Rachel Zoubrinetzky, Ahmed Zaher, Sylviane Valdois

**Affiliations:** ^1^Department of Psychology, Faculty of Arts, Al-Qadisiyah University, Al Diwaniya, Iraq; ^2^Center Référent des Troubles du Langage et des Apprentissages, Pôle Couple-Enfant, Center Hospitalier Universitaire, Grenoble, France; ^3^Université Grenoble Alpes, Université Savoie Mont Blanc, CNRS, LPNC, Grenoble, France

**Keywords:** Arabic reading, reading fluency and comprehension, visual attention span, phonological awareness, vowelized script, non-vowelized script

## Abstract

**Introduction:**

Visual attention span is a measure of multielement parallel processing. Individuals with higher visual attention span are expected to allocate more attention to letters within strings, which boosts letter identification and translates into more efficient reading. Given the high visual complexity of the Arabic writing system, we expected visual attention span to be an important predictor of reading in the Arabic language.

**Methods:**

Native Arabic readers from Grade 4 and Grade 5 were recruited in Iraqi schools. We assessed the contribution of visual attention span to their reading fluency performance in tasks of fully vowelized word and pseudo-word reading, non-vowelized text reading, and written text comprehension. Their phonological awareness, IQ, and single letter processing speed were further evaluated.

**Results:**

Results showed that visual attention span was a significant unique predictor of all the reading measures. Visual attention span and phonological awareness accounted for a similar amount of variance in word and pseudo-word reading fluency. Visual attention span was a far higher predictor than phonological awareness for text reading fluency and the sole predictor of text comprehension.

**Discussion:**

The role of visual attention span to reading is discussed by reference to current word recognition models. Higher involvement of visual attention is expected in vowelized script to compensate for increased crowding in the presence of diacritics. Visual attention would thus contribute to sub-lexical orthographic parsing and favor orthography-to-phonology mapping, in particular for the pseudo-words that do not benefit from efficient lexical feedback. In non-vowelized script, higher visual attention would enhance the accurate and fast identification of root letters within words, thus resulting in faster word recognition.

## Introduction

There is growing evidence that phonological awareness (PA) and visual attention span (VAS) independently contribute to explain inter-individual variations in reading outcomes (Valdois et al., 2019a; Perry and Long, 2022). PA reflects the capacity to identify and manipulate phonological units (like phonemes, rimes, or syllables) within spoken words. Higher PA is expected to contribute to efficient orthography-to-phonology mapping at the sublexical level, which would promote novel word (or pseudo-word) decoding and contribute to word-specific orthographic knowledge acquisition for fast word recognition (Share, 1999; Ziegler et al., 2014; Castles et al., 2018; Pritchard et al., 2018). VAS is a measure of multi-element parallel processing skills in the visual modality. It is defined as the number of distinct elements that can be simultaneously processed in a visual array and depends on the amount of visual attention available for processing (Bosse et al., 2007; Frey and Bosse, 2018; Valdois, 2022). Higher VAS reflects the fact that a higher amount of visual attention capacity is deployed for letter identification within strings, leading to process more letters simultaneously. This ability is thought to support the processing of orthographic chunks as wholes, which boosts reading fluency (Lallier and Carreiras, 2018; Valdois et al., 2019a). Beyond PA, the involvement of VAS to reading has been mainly studied in Western European languages, thus in alphabetic languages that differ in orthographic transparency, but use a small inventory of relatively simple characters (mainly, Latin letters) to transcribe spoken words (Verhoeven and Perfetti, 2021). Modulation of reading performance by VAS might differ in the languages that use more complex written characters, and for which character identification is more attention-demanding. Recent studies have shown that VAS is predictive of reading in Chinese, a language that uses a large inventory of complex characters (Zhao et al., 2017, 2018; Huang et al., 2019; Chan and Yeung, 2020; Cheng et al., 2021). However, the concurrent involvement of PA was not examined in most of these studies and when it was, inconsistent findings were reported (Zhao et al., 2018; Cheng et al., 2021). The present study focuses on the Arabic language, a Semitic language that is particularly challenging for the visual system due to the high visual complexity of its characters and the use of a cursive script so that individual characters are not well segregated within words (Verhoeven and Perfetti, 2021). Our main purpose was to determine whether PA and VAS are concurrent predictors of reading fluency in Arabic and whether VAS might contribute to Arabic reading more substantially than PA, due to the visual complexity of its writing system.

### The Arabic orthography

Arabic orthography is characterized by high visual complexity. First, many letters share the same basic shape and only differ by the number and location of dots associated with this basic shape (e.g., ب ث ت; Saiegh-Haddad and Henkin-Roitfarb, 2014). These letters are very similar graphically, which makes letter processing (i.e., letter detection, recognition, or identification) difficult, either presented in isolation or within strings (Ibrahim et al., 2002; Eviatar et al., 2004; Abdelhadi et al., 2011; Eviatar and Ibrahim, 2014). Second, Arabic is written in a cursive script, so that letters can ligate to the preceding or following letter. However, the combination of position and ligation changes the form of many letters. Thus, most Arabic letters change in shape depending on whether they appear at the beginning, middle, or end of a word. As a result, letter processing is a challenge for beginning, or even more advanced, native Arabic readers (Verhoeven and Perfetti, 2021).

Word recognition is also challenging for the visual system. Arabic words are composed of a root and a pattern morpheme. Roots are typically made of three consonants that convey the core meaning of the word, while patterns are primarily vocalic, corresponding to long vowels (sometimes augmented with certain consonants) that convey morphosyntactic and phonological information. Precise encoding of both the identity and relative position of root letters is critical for word processing in Arabic, as several different roots share the same letters but in a different order (Frost, 2012). The consonantal root letters combine with the word pattern to derive content words (verbs and nouns). However, morphology is non-concatenative. Arabic words are always composed by intertwining root-morphemes with word-pattern morphemes. For example, the three consonantal root-morpheme “k-t-b” when combined to the word-pattern “CaCiC” derives the word “katib” (writer) but combination with the pattern “maCCuuC” derives the word maktuub (written). Thus, word processing requires the orthographic processing system to pick up precise information on the identity and relative order of root letters that can be dispersed within the word in many different positions. This is particularly challenging for the visual system given that fast root processing is critical for efficient word recognition (Velan and Frost, 2011; Perea et al., 2014; Shalhoub-Awwad and Leikin, 2016).

Moreover, each word can be written using two orthographic versions of the Arabic script. In fully vowelized script, short vowels are indicated using diacritics that appear below or above the letters within the whole pattern of the written word. Indeed, the addition of vocalic patterns to the consonant letters of the root only provides partial phonological information on word pronunciation. Diacritics complement this information, yielding to infer a unique pronunciation of the written word. The vowelized script is mainly used in children books at the beginning of literacy instruction. In the non-vowelized script, diacritics are omitted, which inflates the number of homographs and makes decoding heavily dependent on context. The two scripts differently tax the cognitive system. In fully vowelized script, the use of diacritics is useful in facilitating phonological processing, but addition of the diacritic marks increases words’ graphical complexity which additionally tax visual processing. Thus, the addition of short vowels enhances reading accuracy in beginning readers (Abu-Rabia, 1997, 2001) but further increases processing time (Roman and Pavard, 1987; Saiegh-Haddad and Schiff, 2016). Faster reading is typically reported for non-vowelized words (Ibrahim, 2013; Taha, 2016; Abu-Liel et al., 2021) but efficient reading then relies on the processing of larger (morphological) units (Frost, 2005) and is more dependent on context (semantic and syntactic information).

### The cognitive processes involved in reading

It is well established that learning to read builds upon PA (Ehri et al., 2001; Melby-Lervåg et al., 2012), and that PA is important for reading acquisition across languages (Caravolas et al., 2013; Moll et al., 2014). Although the vast majority of research on the role of PA on reading acquisition has been undertaken in Western European languages (Share, 2008), a growing number of studies supports the involvement of PA in reading in other language families (for a review in Chinese, see Song et al., 2016). With respect to the Arabic language, the PA-reading relationship was consistently reported in both vowelized and non-vowelized script (Abu-Rabia et al., 2003; Elbeheri and Everatt, 2007; Smythe et al., 2008; Taibah and Haynes, 2011; Farran et al., 2012; Abu Ahmad et al., 2014; Asaad and Eviatar, 2014; Ghanem and Kearns, 2015; Tibi and Kirby, 2018, 2019).

Beyond phonology, reading also involves visual mechanisms for the accurate identification of letters within strings. Current word recognition models make clear statements about the mechanisms at stake (Norris, 2013; Phénix et al., 2016). These models postulate a first level of visual feature detection for letter identification. The letters that share more visual features are more prone to be confused with one another, so that their accurate identification requires longer processing time. Successful letter identification is thus more demanding in languages, like Arabic, that use a set of visually complex letters, many of which share high visual similarity (Pelli et al., 2006; Boudelaa et al., 2020). Letter visibility within strings is further modulated by visual acuity (Nazir et al., 1991; Whitney, 2001) and lateral interference between letters, i.e., crowding (Pelli et al., 2007; Norris and Kinoshita, 2012). Letter identification decreases with eccentricity (i.e., distance of the letter from gaze position) due to the limits imposed by visual acuity. It is further affected by crowding effects, the fact that identification is degraded by the proximity of adjacent letters (Bouma, 1970; Martelli et al., 2009; Whitney and Levi, 2011). Although visual acuity is not sensitive to the orthographic system properties, crowding effects might be more detrimental in a language like Arabic, in which most letters are connected through ligature within words. Further, crowding might affect letter processing in vowelized script more than in non-vowelized script, due to the presence of additional diacritic marks (Hermena et al., 2015).

Finally, letter identification within strings is affected by visual attention (Lien et al., 2010; Waechter et al., 2011). Recent models of word recognition assume that visual attention acts as a filter that enhances letter identification under the attentional focus (Phénix et al., 2018; Ginestet et al., 2019; Valdois et al., 2021a). Visual attention is then conceived as a Gaussian distribution that deploys over the word letter string. The letters that receive more attention are more accurately and faster identified, which at least in part counter-balances the detrimental effects of poor letter discriminability, low visual acuity, and crowding. Thus, visual attention might be particularly relevant to explain inter-individual variability in learning to read in Arabic.

The measure of visual attention span (VAS) is typically used in behavioral studies to estimate the amount of visual attention available for letter string processing (Valdois, 2022). Children with higher VAS read more accurately and faster than children with lower VAS (Bosse et al., 2007; Bosse and Valdois, 2009; Zoubrinetzky et al., 2014, 2016; Valdois et al., 2021b) and VAS abilities measured prior to literacy instruction predict later reading skills (Valdois et al., 2019a). Significant involvement of VAS on reading has been reported in a variety of languages, like English (Bosse et al., 2007; Chen et al., 2016; Cirino et al., 2022), Brazilian Portuguese (Germano et al., 2014), Spanish (Lallier et al., 2014), Greek (Niolaki and Masterson, 2013), Dutch (van den Boer et al., 2013, 2014, 2015; Van den Boer and de Jong, 2018), or Chinese (Zhao et al., 2017, 2018; Chen et al., 2019; Huang et al., 2019; Cheng et al., 2021). Importantly, the contribution of VAS to reading achievement has been found independent of the effects of PA in both typical (Bosse and Valdois, 2009; van den Boer et al., 2013, 2015; Van den Boer and de Jong, 2018; Valdois et al., 2019a) and dyslexic/poor readers (Bosse et al., 2007; Germano et al., 2014; Zoubrinetzky et al., 2014, 2016; Chen et al., 2016; Valdois et al., 2021b). Evidence that VAS and PA are independent cognitive skills is further supported by neurobiological studies showing that VAS relies on the activation of brain regions, the superior parietal lobules, that belong to the dorsal attentional brain network and differ from those involved in PA and oral language tasks (Peyrin et al., 2011, 2012; Lobier et al., 2012a, 2014; Reilhac et al., 2013; Valdois et al., 2019b; see also Liu et al., 2022).

The few studies that investigated the VAS-reading relationship in Arabic readers reported modulations of VAS due to the Arabic orthography constraints and variations of the VAS-reading relationship depending on the Arabic script (Awadh et al., 2016; Lallier et al., 2018). Awadh et al. (2016) measured VAS abilities in highly educated Arabic, French, and Spanish adult readers through standard five-letter report tasks (Valdois, 2022). Despite matching for physical length (thus, visual acuity) and control for crowding, Arabic readers exhibited lower VAS than French or Spanish readers. This suggests that letter identification may be more attention demanding in Arabic, due to the visual complexity of letters, so that lesser letters would be simultaneously identified within strings. However, Awadh et al. (2016) reported no significant correlation between VAS performance and text reading fluency in their highly educated Arabic participants. Lallier et al. (2018) hypothesized that the VAS-reading relationship may vary depending on the Arabic script. They administered a visual one-back VAS task to Grade 4 native Arabic readers who were asked to read the same texts in either the vowelized and non-vowelized script. Results showed no relationship of VAS with text reading, whatever the script. However, a relationship emerged in the subgroup of Arabic children who were more proficient in non-vowelized than in vowelized text reading. Although the interpretation of these findings is not straightforward, they might suggest a higher development of VAS in children who are better at reading non-vowelized texts. Overall, only a couple of studies have investigated the potential contribution of VAS to reading performance in the Arabic language. The contribution of PA is more documented but no study explored the concurrent effects of PA and VAS on reading skills in Arabic.

### The present study

Our aim in the current study was to examine the unique contribution of VAS to reading skills (word, pseudo-word, and text reading) in monolingual native Arabic children, after control of PA. We expected that variations in VAS would constrain the number of characters (letters and/or diacritics) that would be simultaneously identified within the written string, thus contributing to reading fluency, independently of PA. Although both PA and VAS were expected to relate to reading performance, we anticipated that the magnitude of the relationship would vary depending on the reading subskills and Arabic script. Assuming that pseudo-word reading relies more on phonological decoding than word (or text) processing and that PA is involved in the acquisition of mappings between sub-lexical orthographic and phonological units, we expected PA to contribute more to pseudo-word than word or text reading. In contrast, the reading of non-vowelized texts should rely more on lexical (morphological and semantic) knowledge through the processing of larger orthographic units, a condition that would be more demanding on VAS than PA skills. We further examined whether and to what extent VAS and PA predicted unique variance in non-vowelized text comprehension. Assuming that reading in non-vowelized script involves root morpheme identification for word core meaning processing and reliance on contextual information (thus relying on orthographic chunks); VAS was expected to further predict text comprehension while PA might less strongly contribute, if any.

## Materials and methods

### Participants

One hundred and thirty-four monolingual native Arabic speakers from Grade 4 and Grade 5 were recruited in six primary schools of the Babylon area in Iraq. In Iraqi schools, children are exposed to vowelized orthography during the first 2 years of literacy instruction. They are familiarized with the non-vowelized script in Grade 3 and almost exclusively confronted to non-vowelized materials in later grades. Thus, Grade 4–5 participants were expected to have good reading expertise in non-vowelized script while remaining sufficiently familiar with the vowelized script. Twenty outliers were detected using the Mahalanobis robust distance (Minimum Covariance Estimation; Leys et al., 2018), so that the sample size was reduced to 114 students (62 males). The participants had a mean age of 124 months (SD = 4 months). All of them had normal audition and normal or corrected-to-normal vision. They were reported to attend school regularly and had no history of neurological illness or brain damage. Their general cognitive abilities were tested by a fluid intelligence test, the Progressive Matrices Standard (version for Arab populations: Hammadi, 2012), showing a mean score of 26.82 (SD = 5.64). Official authorizations from the Iraqi ministry were obtained for experimentation at school together with written informed consent from each child legal guardians.

### Measures

The test session included reading tasks of word and pseudo-word reading in vowelized script and tasks of non-vowelized text reading for the estimation of reading fluency and written text comprehension. Two phonological awareness tasks of rhyme judgment and phoneme deletion, and two VAS tasks of whole and partial letter report were further administered together with a control task of single letter identification threshold. All tasks were created for the experiment^1^. The children were tested individually in a quiet room of their school.

#### Reading assessment

##### Text reading

The children were asked to read aloud a text that was entitled: “The Beautiful Butterfly and the Little Child.” The text was proofread by Iraqi linguists from al Qadisiyah University and the University of Babylon who checked that the language level used in the text was appropriate for fourth and fifth grade readers. The text consisted of 181 words, most of which were non-vowelized except for a few words which required diacritics to resolve semantic ambiguity. The text was presented in black on a white sheet accompanied by colored drawings. Participants were asked to read the text aloud as quickly and accurately as possible. Reading was stopped after 2 min or the reading time recorded if lower than 2 min. Text reading fluency was computed for each participant as the number of words accurately read per minute.

##### Word reading

In the absence of resources on Arabic word frequency for children in Iraq, we created a database of the words which children were exposed to during the three first years of literacy instruction. The database provided the number of occurrences of each vowelized word together with their length. Forty vowelized words were selected that varied in length from 3 to 8 letters and were randomly chosen in the different quartiles of occurrence. The words had an average length of 5.05 letters (SD = 1.63) and included 2.3 (SD = 1.7) diacritics on average. They had an orthographic frequency of 145.20 per million on average, according to the ARALEX database (Boudelaa and Marslen-Wilson, 2010). The list of words is provided in Appendix. The words were presented listed in column, one word below the other, printed in black on a white sheet. The children were asked to read the words aloud as accurately and as quickly as possible. Reading time and reading accuracy were recorded. The number of words correctly read per minute was calculated for each participant.

##### Pseudo-word reading

A list of 20 pseudo-words was created for the purpose of the study. The pseudo-words were derived from real words by changing the location of two letters to construct a new pronounceable letter string that included at least one non-existing root or pattern (e.g., the pseudo-word وَلْصَنا /wals’ana/was built from the word وَصَلْنا/was’alna/). All pseudo-words were written with diacritics (i.e., vowelized). They had an average length of 5.10 (SD = 0.88) letters, ranging from 3 to 6 letters, and included 2.95 diacritics (SD = 0.78) on average. The pseudo-words were presented in column, one below the other, printed in black on a white sheet. The children were warned that the items to be read were invented words and they were asked to read them aloud as accurately and as quickly as possible. Pseudo-word reading fluency was computed for each participant as the number of pseudowords accurately read per minute.

##### Text reading comprehension

Three short stories were taken from websites offering stories for children. The texts written without diacritics were adapted to the comprehension level of 10–11 years-old students. They were submitted to specialists of the Arabic language to verify their relevance and linguistic integrity. The children were asked to read each text silently. Each text was followed by six questions and a multiple choice between four possible responses. The questions were of three types: (1) the easier ones required searching for a word or part of a sentence that was explicitly provided in the text (four questions for the first text, one question for the second and one for the third); (2) a second set of questions required making inferences from the text, thus relying on more in-depth analysis of the text meaning (one question for the first text, two for the second and three for the third); and (3) the third set required a good comprehension of the whole text making the child able to choose the title that best summarized the whole text meaning (one question for each text). The number of correct responses for the six questions of each of the three texts was recorded (max = 18).

#### Phonological awareness

The words used in the two phonological tasks were extracted from the children reading books. A composite score was created by addition of scores on the two phonological tasks (max = 31).

##### Rhyme oddity detection

At each trial, the participant heard three spoken words, all but one of which shared a common rhyme. The participant had to detect the odd word. For example, the child was asked which word was the odd one among “زين (zyn)-عين (Eyn)-بَزاز (bzAz).” (expected response: بَزاز/bzAz). All three words were short and of high frequency. The position of the odd word was randomly varied through the different trials. The 16 trials were preceded by four training trials for which children received feedback. The dependent variable was the number of odd words accurately identified (max = 16).

##### Phoneme deletion

A spoken word (5.8 phoneme-long on average, from 3-to-8 phonemes) was orally pronounced by the examiner followed by a phoneme. The child had to mentally remove the phoneme and respond saying what was left. For example, the child was asked: “What is أليفة (Alyfp), if you remove the/f/? The phoneme to be deleted was randomly located in the initial, medial, or final part of the word. Fifteen target words were presented, preceded by a six-word training session. The dependent variable was the number of correct responses (max = 15).

#### Assessment of visual attention span and single letter identification

Two tasks of whole and partial letter report were used to assess VAS abilities. A task of single letter identification threshold was further administered to control for single letter processing speed. The letter report tasks were displayed on a PC computer using E-prime software (E-prime Psychology Software Tools Inc., Pittsburgh, United States). A preliminary study carried out on an independent group of 15 participants revealed that their performance was very low when confronted to strings of five Arabic letters, the string length typically administered to evaluate VAS skills using Latin letters. As a result, and based on evidence that even adult skilled readers could only process an average of 3.68 out of five Arabic letters when briefly presented within VAS tasks (Awadh et al., 2016), the two tasks of whole and partial report were administered using strings of four Arabic letters.

##### Whole and partial report

###### Stimuli

Ten consonants were selected from the 28 letters of the Arabic alphabet (ث/ح/د/ع/ف/ص/ط/ك/ھ/ي/). The set of consonants was chosen to include only one copy of each of the basic forms of Arabic letters. Thus, only one of the letters that shared the same basic shape was selected (for example, we only selected the leftmost from the three following letters, ب, ت, ث). Random four letter-strings were then built up from the 10 consonants. The strings contained no repeated letters. The four-consonant strings never matched the root or the pattern of a real word. The letters were displayed in black on a white background. Each character string subtended an angle of 4.2° (7 mm high) with a distance of 0.57° between the edges of each character to minimize crowding effects. Twenty four-letter strings were displayed in the whole report condition, 40 in partial Report.

###### Procedure

**Figure 1:**
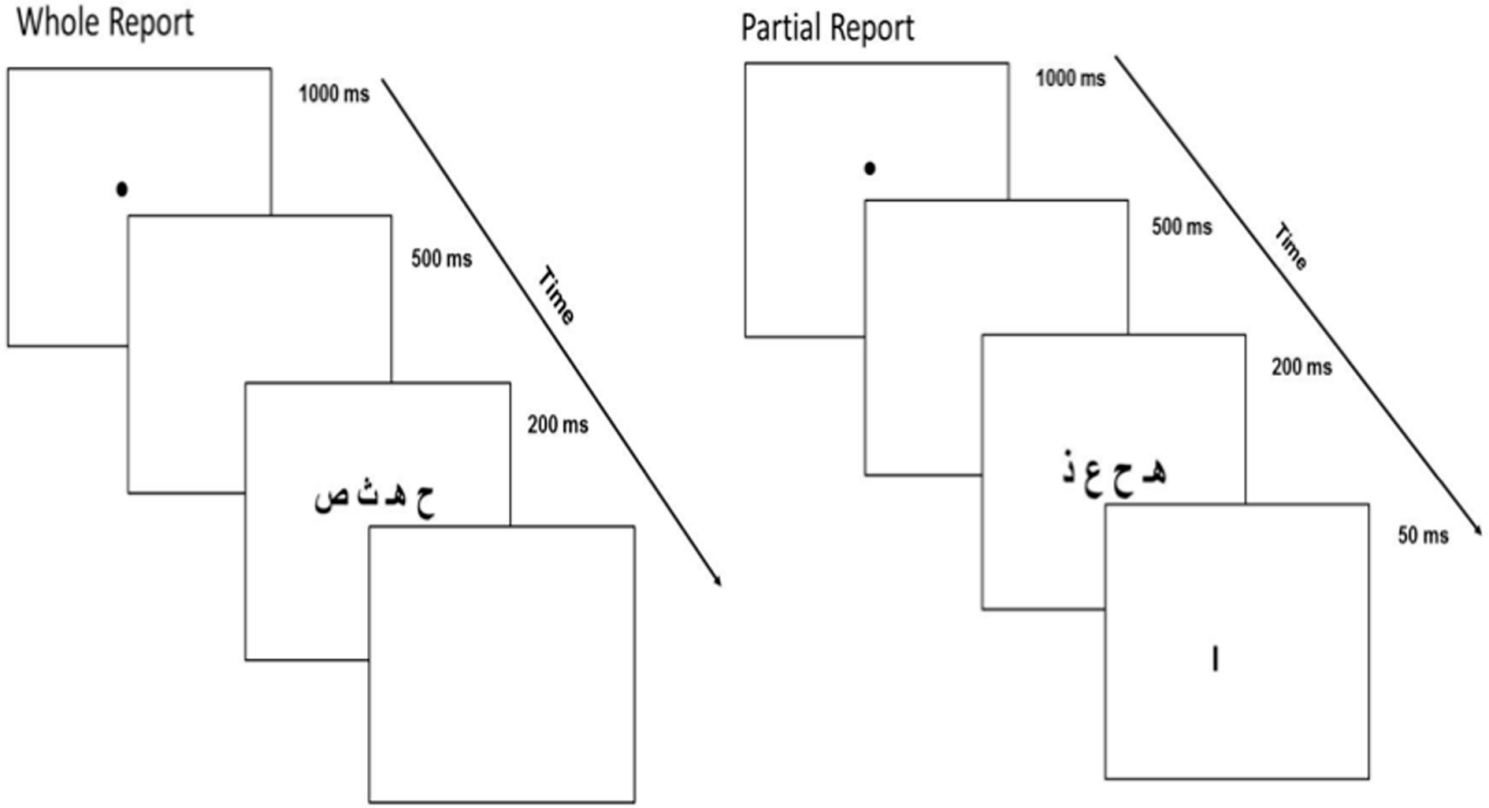
Illustration of the whole and partial report tasks using Arabic letters.

At the beginning of each trial, a central fixation point was presented for 1,000 ms followed by a blank screen for 500 ms. Then, a letter-string was displayed centered on the fixation point for 200 ms, a presentation duration long enough for an extended glimpse, yet too short for a useful eye movement. In the whole report task, children had to report verbally as many letters as possible immediately at the offset of the string. In partial report, a vertical bar indicating the location of the letter to be reported was displayed 1.1° below the target letter, immediately after the letter-string disappeared. Participants were asked to report the cued letter only. In both tasks, the experimenter pressed a button to start the next trial after the participant’s oral response. The experimental trials were preceded of 10 training trials for which participants received feedback. No feedback was given during the experimental trials. The dependent measure was the number of letters accurately reported (identity, not location) across the 20 trials in whole report (max = 80) or across the 40 trials in partial report (max = 40). To balance the contribution of each task, a VAS composite score (expressed as a percentage) was computed using the following relation: Composite VAS_score_ = (Global_score_ + 2 × Partial_Score_) × 100/2 × 80. An illustration of the global and partial report tasks is provided in Figure 1.

##### Letter identification processing efficiency

To control for single letter processing skills, each of the 10 letters used in the VAS report tasks were randomly presented (five times each) with the same physical characteristics as in the VAS tasks. Presentation duration was varied (33, 50, 67, 84, and 101 ms) so that each letter appeared once at each presentation duration. At the offset of the letter, a mask (13 mm high, 37 mm wide) was displayed for 150 ms. Participants were asked to name the letter immediately after its presentation. The test trials were preceded of 10 practice trials (two for each presentation duration) for which participants received feedback. The identification threshold was then calculated for each child as the minimum presentation duration that yielded at least 80% accurate identification.

## Results

### Descriptive statistics

Table 1 provides descriptive statistics of participants’ performance for all the predictive variables and reading outcomes. Scores on reading fluency, VAS, and text comprehension were normally distributed. As shown on Table 1, raw scores on the phonological awareness tasks were relatively high, with a mean performance of 13.17 out of 15 on the phoneme deletion task and of 13.84 out of 16 on the rhyme oddity detection task. As the normality assumption was not verified on the measures of phonological awareness, the Yeo-Johnson transformation (Yeo and Johnson, 2000) was used to ensure symmetry of the distributions for these variables. On average, 60.61 letters were accurately reported in the whole report VAS task, suggesting that three out of the four letters were identified on average at each trial. The letter identification threshold measure showed that a presentation duration of 94.5 ms (ranging from 52 to 133 ms) on average was required for the accurate identification of at least 80% isolated letters.

**Table 1:** Means and standard deviations (SD), Median, Minimum (min), and Maximum (max) scores for the whole measures of IQ, single letter identification threshold, reading, phonological awareness (PA), and visual attention span (VAS).

	Mean	SD	Median	Min	Max
IQ	26.82	5.64	27.00	8.00	36.00
Identification threshold	94.50	21.31	97.50	52.00	133.00
*Reading tasks*					
Long words (wpm)	14.32	6.04	13.04	1.71	34.29
Short words (wpm)	17.44	6.50	15.48	6.36	38.71
Pseudo-words (wpm)	12.25	4.96	11.89	0.00	26.15
Text reading (wpm)	95.52	44.17	95.50	27.00	172.00
Text comprehension (/18)	14.20	2.87	14.00	8.00	18.00
*Phonological awareness*					
Phoneme deletion (/15)	13.17	2.48	14.00	5.00	15.00
Rhyme oddity (/16)	13.84	2.52	15.00	7.00	16.00
PA composite score	27.01	4.42	29.00	14.00	31.00
*Visual attention span*					
Whole report (/80)	60.61	9.39	62.00	40.00	79.00
Partial report (/40)	29.18	5.32	28.50	17.00	39.00
VAS composite score	89.79	13.67	92.00	57.00	114.00

### Correlation analyses

Simple and partial correlation coefficients (after controlling for the effect of IQ) between all the measures are reported in Table 2. As shown on Table 2, all the measures corresponding to the same construct were positively and highly correlated (all ps < 0.001), suggesting good between-test reliability. Correlation coefficients close to 0.60 were found among the different reading tasks (from 0.58 to 0.62). The two measures of phonological awareness correlated at 0.57, thus justifying computation of a composite score as the sum of performance on the two tasks. In the same way, a composite weighted VAS score was computed from scores on the two tasks of whole and partial report that correlated at 0.69.

**Table 2:** Pearson’s correlation coefficients (above the diagonal) and partial correlations (below the diagonal) after control of IQ (adjusted using a Bonferroni correction).

	**2**	**3**	**4**	**5**	**6**	**7**	**8**	**9**	**10**	**11**	**12**
1. IQ	0.19	0.28	0.27	0.23	0.26	0.05	0.18	0.31	0.19	0.25	0.27
2. Word reading (wpm)	-	0.64^***^	0.60^***^	0.30	0.39^**^	0.24	0.36^*^	0.33^*^	0.49^***^	0.33	0.45^***^
3. PW reading (wpm)	0.62^***^	-	0.65^***^	0.30	0.54^***^	0.34^*^	0.50^***^	0.52^***^	0.59^***^	0.28	0.60^***^
4. Text reading (wpm)	0.58^***^	0.62^***^	-	0.52^***^	0.39^**^	0.37^**^	0.43^***^	0.79^***^	0.79^***^	0.52^***^	0.86^***^
5. Comprehension	0.26	0.25	0.49^***^	-	0.21	0.18	0.22	0.53^***^	0.31	0.22	0.45^***^
6. Phoneme omission	0.36^**^	0.50^***^	0.34^*^	0.16	-	0.56^***^	0.88^***^	0.20	0.33^*^	0.28	0.29
7. Rhyme judgment	0.24	0.34^*^	0.37^**^	0.17	0.57^***^	-	0.89^***^	0.27	0.29	0.26	0.30
8. Phono composite score	0.34^*^	0.47^***^	0.40^**^	0.19	0.88^***^	0.89^***^	-	0.26	0.35^*^	0.31	0.34^*^
9. VAS whole report	0.29	0.47^***^	0.78^***^	0.50^***^	0.12	0.27	0.22	-	0.70^***^	0.42^***^	0.91^***^
10. VAS partial report	0.47^***^	0.57^***^	0.78^***^	0.28	0.30	0.28	0.33^*^	0.69^***^	-	0.48^***^	0.93^***^
11. Identification threshold	0.29	0.23	0.49^***^	0.18	0.23	0.25	0.28	0.37^**^	0.45^***^	-	0.49^***^
12. VAS composite score	0.42^***^	0.57^***^	0.85^***^	0.41^***^	0.24	0.30	0.30	0.91^***^	0.93^***^	0.45^***^	-

***p < 0.001; **p < 0.01; *p < 0.05.

More interesting for the present purpose, the composite measures of VAS and PA correlated significantly with all the reading fluency measures, except for PA and text reading comprehension. Children with higher PA showed higher reading fluency; those with higher VAS exhibited better performance in both reading fluency and text comprehension. Moreover, as expected assuming that PA and VAS tap different cognitive skills, none of the VAS measures significantly correlated with any of the PA measures.

### Regression analyses

Regression analyses were conducted to explore the unique contribution of VAS to reading fluency and text comprehension. We used the R stats package within the R environment (R core development team, 2020) for statistical computing to run linear regressions. Four regression models were computed, one for each of the reading outcomes, namely word and pseudo-word reading fluency, text reading fluency, and text comprehension. The effects of grade level (Grade 4 and Grade 5), IQ, and letter identification threshold were controlled for in all four models. Table 3 presents the unique contribution of VAS and PA (and the control variables) to the different reading outcomes.

**Table 3:** Predictors of the reading outcomes.

Dependent variables	Word reading (wpm)			PW reading (pwpm)			Text reading (wpm)			Text comprehension		
*Equation results:*	*R* = 0.266; Adj.*R*^2^ = 0.232; *F*(5,108) = 7.828^***^			*R* = 0.477; Adj.*R*^2^ = 0.452; *F*(5,108) = 19.680^***^			*R* = 0.776; Adj.*R*^2^ = 0.765; *F*(5,108) = 74.7^***^			*R* = 0.236; Adj.*R*^2^ = 0.201; *F*(5,108) = 6.67^***^		
*Predictors*	*β*	*t*	*∆R^2^*	*β*	*t*	*∆R^2^*	*β*	*t*	*∆R^2^*	*β*	*t*	*∆R^2^*
Grade level	0.163	0.934	0.008	−0.057	−0.386	0.001	0.198	2.044	0.037^*^	0.300	1.680	0.025~
IQ	0.038	0.439	0.002	0.112	1.529	0.021	0.015	0.321	0.001	0.101	1.143	0.012
Identification threshold	0.110	1.123	0.115	−0.102	−1.226	0.014	0.125	2.291	0.046^*^	0.009	0.088	<0.001
PA composite score	0.206	2.293	0.046^*^	0.340	4.484	0.157^***^	0.124	2.505	0.055^*^	0.050	0.543	0.003
VAS composite score	0.293	2.882	0.071^**^	0.515	6.004	0.250^***^	0.724	12.888	0.606^***^	0.358	0.543	0.099^***^
Constant	−0.075	−0.652	-	0.026	0.269	-	−0.090	−1.427	-	−0.14	−1.172	-

***p < 0.001; **p < 0.01; *p < 0.05.

The whole model accounted for 23.2 and 45.2% of variance, respectively, for word and pseudo-word reading fluency, 76.6% of variance in text reading fluency, and 20.1% in written text comprehension. As can be seen in Table 3, the unique contribution of VAS to reading fluency was significant for all tasks, showing that higher VAS was associated with more proficient reading fluency. VAS contribution was particularly high for text reading fluency, accounting for 60.6% of unique variance. For pseudo-word fluency, VAS accounted for 25% of variance, while its contribution was relatively low for word reading fluency (7.1% of explained variance). PA was another unique predictor of performance in word, pseudo-word and text reading fluency.

Visual attention span and PA contributed to explain a similar amount of variance in both pseudo-word (25 vs. 16%, *F*_(1, 108)_ = 2.01, *p* = 0.160) and word (7 vs. 5%, *F* < 1, ns) reading fluency. In contrast, the predictive power of VAS was stronger than that of PA in text reading fluency (60 vs. 6%, *F*_(1, 108)_ = 54.96, *p* < 0.001) and VAS was the sole predictor of text reading comprehension.

## Discussion

The present study investigated VAS skills in native Arabic children to determine whether they uniquely influenced reading fluency and reading comprehension in Arabic. For this purpose, standard VAS tasks of whole and partial letter report were administered to Grade 4–5 native Arabic children; their reading skills were evaluated through tasks of single word, pseudo-word and text reading fluency, and a task of written text comprehension. The participants’ ability to efficiently identify isolated letters was further estimated to control for potential effects of variations in single letter processing on the VAS-reading relationship. The overall findings argue for an independent influence of VAS in both reading fluency and reading comprehension. PA was an additional unique predictor of the reading fluency measures but did not influence reading comprehension.

The standard paradigms of whole and partial letter report were used to estimate VAS abilities but Latin letters were replaced by Arabic letters in the present study. In these tasks, performance primarily reflects the amount of visual attention available for multiletter parallel processing. Although standard paradigms require the verbal report of letter names, previous studies did not support a visual-to-phonological mapping account of VAS performance (for a review, see Valdois, 2022). Performance across VAS tasks is highly correlated either using verbal or non-verbal material (Lobier et al., 2012a; Chan and Yeung, 2020), and the same attentional brain regions are activated regardless of the verbal or non-verbal nature of the stimuli (Lobier et al., 2012b, 2014). Moreover, if phonologically-driven, VAS performance would likely relate to phonological skills, which was not previously reported (Valdois, 2022) and not found in the present study. Lexical effects on VAS performance are further prevented by the use of random consonant strings. In the VAS Arabic version, the strings did not include any existing root or pattern morpheme, so that letter identification did not benefit from lexical feedback but was mainly visually-driven. According to visual word recognition models, letter identification within string is modulated by visual acuity, crowding and the amount of visual attention available for processing. It is further dependent on letter discriminability (i.e., to what extent each target letter shares features with concurrent letters of the same alphabet). Inter-character spacing is systematically increased in VAS tasks to avoid crowding effects, so that inter-individual variations in performance cannot be attributed to differences in crowding. Visual acuity is expected to be constant, as far as strings do not vary in length and participants have normal or corrected vision. Thus, performance on VAS tasks mainly reflects how visual attention and letter discriminability interact for the accurate identification of letters within strings. Inter-individual differences in Arabic letter discriminability were estimated through the task of single letter identification threshold. Results showed high inter-individual variations in single letter processing skills. Furthermore, single letter identification efficiency correlated with performance on VAS tasks and text reading fluency. To zeroing on the impact of visual attention on reading, the VAS-reading relationship was studied while systematically controlling for inter-individual differences in single letter processing.

Current results showed that, beyond PA, VAS uniquely predicted Arabic word and pseudo-word reading fluency. This is well in line with evidence from European languages that VAS independently contributes to both word and pseudo-word reading (Bosse et al., 2007; Bosse and Valdois, 2009; Lallier et al., 2014; van den Boer et al., 2015; Valdois et al., 2019a, 2021a). An involvement of PA to Arabic word and pseudo-word reading was previously reported and PA is considered as a strong predictor of reading performance in Arabic (Saiegh-Haddad and Geva, 2008; Taibah and Haynes, 2011; Farran et al., 2012; Abu Ahmad et al., 2014; Tibi and Kirby, 2018, 2019). The present findings show that the contribution of VAS was equivalent to that of PA on the two measures of vowelized word and pseudo-word reading fluency. Reading single words and pseudo-words written in vowelized script relies on the mapping between sub-lexical orthographic and phonological units, in particular for pseudo-word processing that does not benefit from lexical feedback. Successful mapping is facilitated when sub-lexical phonological units are successfully identified within spoken words due to efficient PA skills. However, the contribution of VAS suggests that visual attention was involved in the successful identification of relevant orthographic units, in particular for pseudo-word processing. The processing of Arabic pseudo-words is particularly taxing for the visual system. In the absence of helpful lexical feedback, accurate letter identification almost exclusively relies on bottom-up sensory information. But extraction of letter identity sensory information is degraded due to high confusability between Arabic letters and increased crowding in the presence of diacritic marks. Moreover, efficient processing of the small superscripted marks that represent short vowels (i.e., the diacritics) is in particular critical for pseudo-word reading. The pronunciation of letters is ambiguous in the absence of diacritics, so that letters and diacritics have to be simultaneously processed for unambiguous orthography-to-phonology mapping. Visual attention is known to improve discriminability and accelerate information processing (Carrasco and McElree, 2001), two properties that would contribute to enhance letter and sublexical orthographic unit processing within strings. Previous findings suggested a contribution of VAS to graphemic parsing in European languages (Zoubrinetzky et al., 2014). More generally, VAS might be involved in the identification and segregation of relevant sub-lexical orthographic units for their mapping with phonology. It has been previously argued that individuals with higher visual attention resources would allocate more attention for the identification of visual characters (letters and diacritics) within letter strings. Assuming that a large amount of visual attention is required for in-depth identification of relevant information in visually complex and crowded environments, and assuming that a fixed amount of attention resources is available for processing, then available resources might only allow the accurate processing of a limited number of visual characters simultaneously, which would predict slow but accurate processing in vowelized script, as typically reported (Roman and Pavard, 1987; Abu-Rabia, 2001; Ibrahim, 2013; Saiegh-Haddad and Schiff, 2016).

The present study further revealed that VAS contributed to explain 60% of unique variance in text reading fluency while PA only moderately contributed. It is widely assumed that, in the absence of diacritics, reading is less reliant on phonological information but more on visual orthographic processing and whole-word recognition (Taouk and Coltheart, 2004; Hansen, 2014). Accurate and fast word recognition then implies fast processing of the consonants that form the root morpheme to favor matching with the corresponding orthographic word representation in long-term memory (Frost, 2005, 2012; Boudelaa, 2014; Perea et al., 2014; Shalhoub-Awwad and Leikin, 2016). One can easily infer that fast identification of root letters dispersed within the word letter-string requires deploying attention over the whole letter string to select relevant information. Individuals with higher visual attention resources (thus, higher VAS) are able to allocate enough attention to more letters within the word string. In non-vowelized Arabic script, this might contribute to accurate and fast identification of root letters among word patterns. Further, higher visual attention resources might favor letter information processing across multiple words in parallel in sentence reading (Snell and Grainger, 2019), which might trigger fast word recognition (Hermena et al., 2021; Khateb et al., 2022).

Last, the present findings argue for an exclusive influence of VAS on written text comprehension in Arabic, as previously reported for the English language (Chen et al., 2016). They are also in line with previous evidence for a non-significant influence of PA on text comprehension in the Arabic language (Elbeheri et al., 2011; Farran et al., 2012). We previously argued that word recognition in text reading was improved when a larger amount of visual attention was allocated to processing. Assuming that text reading comprehension is the product of word recognition and language comprehension (Gough and Tunmer, 1986; Duke and Cartwright, 2021), higher word recognition efficiency due to higher VAS might make more cognitive resources available to built-up and maintain a general model of text meaning. This would predict an indirect contribution of VAS to text reading comprehension. However, text reading comprehension mainly depends on high-level processing skills, like background knowledge and inferencing skills that were not considered in the present study.

Our main contribution in the current study was to provide first evidence that, above and beyond PA, VAS was a unique predictor of reading fluency in the Arabic language. We further argued that referring to theoretical models of word recognition is critical to disentangle the mechanisms involved in visuo-orthographic processing and that such models may be particularly relevant with respect to languages, like Arabic, that are particularly challenging for the visual system. The present findings also open new perspectives for future research. We found that VAS and PA equally accounted for single word and pseudo-word processing in vowelized script while VAS was a stronger predictor of reading fluency for texts written in non-vowelized script. Although some specific features of non-vowelized script may justify higher reliance on visual attention, strong conclusions would require a systematic manipulation of the two scripts. For this purpose, future studies should investigate the relative contribution of PA and VAS to reading performance for similar materials presented in either vowelized or non-vowelized script. The present study revealed only moderate contribution of PA to reading fluency after control of VAS in Grade 4–5 participants. However, PA may contribute more to reading in earlier grades. Investigation of the relative contribution of PA and VAS to reading on a large sample of grades would help better understanding the role of these two skills in reading development in Arabic. Last, the present study focused on PA and VAS as basic predictors of reading development. More research is required to better understanding how these two skills interact with the other predictors of the Arabic language, in particular morphological awareness and morphological processing skills.

## Data availability statement

The raw data supporting the conclusions of this article will be made available by the authors, without undue reservation.

## Ethics statement

The studies involving human participants were reviewed and approved by Official authorizations from the Iraqi ministry. Written informed consent to participate in this study was provided by the participants’ legal guardian/next of kin.

## Author contributions

FA and SV contributed to the conception and design of the study. FA recruited the participants and collected the data. RZ organized the database. AZ performed the statistical analyses. FA, RZ, AZ, and SV wrote sections of the manuscript. All authors contributed to the article and approved the submitted version.

## Funding

FA was supported by a fellowship from the Iraqi Ministry and Campus-France.

## Conflict of interest

The authors declare that the research was conducted in the absence of any commercial or financial relationships that could be construed as a potential conflict of interest.

## Publisher’s note

All claims expressed in this article are solely those of the authors and do not necessarily represent those of their affiliated organizations, or those of the publisher, the editors and the reviewers. Any product that may be evaluated in this article, or claim that may be made by its manufacturer, is not guaranteed or endorsed by the publisher.

## Footnotes

^1^Unfortunately, Cronbach’s alphas are not reported for the different tasks. By-item scores were lost due to technical problems preventing any measure of inter-item homogeneity.
